# Editorial: Stem Cells in Endocrine Tumors

**DOI:** 10.3389/fendo.2021.722790

**Published:** 2021-06-28

**Authors:** Simone Di Franco, Natalia Simona Pellegata, Michaela Luconi, Giorgio Stassi

**Affiliations:** ^1^ Department of Surgical Oncological and Stomatological Sciences, University of Palermo, Palermo, Italy; ^2^ Institute for Diabetes and Cancer, Helmholtz Zentrum München, Helmholtz-Gemeinschaft Deutscher Forschungszentren (HZ), Munich, Germany; ^3^ Department of Biology and Biotechnology "L. Spallanzani", University of Pavia, Pavia, Italy; ^4^ Department of Experimental and Clinical Biomedical Sciences "Mario Serio", University of Florence, Florence, Italy

**Keywords:** endocrine tumor, cancer stem cells (CSCs), biomarker, EMT, tumor metabolism

Endocrine tumors (ETs) include both benign and malignant diseases, originated from the boosted proliferation capacity of the hormone-producing glands of the endocrine system, and are characterized by a high degree of complexity and cellular heterogeneity. In this context, a crucial role in ET initiation and progression seems to be played by cancer cells endowed with stem cell properties, called cancer stem cells (CSCs), only recently identified in ETs, which presence is often associated with chemoresistance and metastatic potential. The aim of this Research Topic is to summarize all the findings regarding the role of CSCs in ETs, the peculiar properties of cancer versus healthy stem cells in different endocrine organs, highlighting the progress made in this research field, as well as the current contradictions. Recent findings have demonstrated that CSCs could arise from normal stem cells, upon acquisition of (epi)genetic hits, or by de-differentiation of progenitor/differentiated cells. For this reason, a better study of CSCs’ healthy counterpart could lead to a better understanding of cancer cell biology and treatment.

In this context, Mariniello et al. study the role of healthy stem cells in the development and self-renewal of endocrine organs (pituitary, adrenal gland, thyroids, parathyroids, gonads, pancreas), as well as their functions in regenerative medicine, highlighting how alterations in the mechanisms acting in stem cells may lead to development of endocrine cancer.

An example of the biological mechanisms underlying transformation of normal to CSCs is reported in the review by Veschi, Verona, Lo Iacono et al. Indeed, despite several thyroid tumorigenesis models have been recently proposed, the thyroid CSC genetic mutation model has been reported to elucidate the molecular mechanisms underlying the distinct thyroid cancer (TC) histopathology and clinical behavior (1). In this review the authors examined the principal pathways and epigenetic alterations sustaining TCSC survival and the complex crosstalk between TCSCs and the tumor microenvironment cell populations, to define more effective therapeutic strategies, as immunotherapy and epigenetic drugs.

In line with the model describing CSCs as derived from healthy stem cells, the review article “Cancer Stem Cells and Neuroblastoma: Characteristics and Therapeutic Targeting Options” Veschi et al. dissected what is already known about the origin and the isolation of CSCs in neuroblastoma (NB), the most common extracranial neoplasm in children. Targeting CSC subset may overcome NB recurrence and resistance to therapy. Of note, the authors discussed the mesenchymal (S-type) and adrenergic (N-type) phenotypes of NB-CSCs with tumor-initiating properties and their epigenetic traits, and explored the most recent therapeutic applications and strategies for CSCs targeting.

A tumor type that shares the same cell of origin as NB is pheochromocytoma, which originates from the chromaffin cells located in the adrenal medulla (pheochromocytoma-PCC) or in parasympathetic paraganglia (paraganglioma-PGL). In their review, Scriba et al. summarize where we stand with the identification of normal chromaffin stem cells (SCs), and of putative CSCs in PCC/PGL. Although the precise role of these cells, characterized by the expression of nestin, Oct4, Nanog, and Sox2, is still unclear, their involvement in the maintenance of stem cell niche, as well as in tumorigenesis is discussed, with particular focus on the development of targeted therapies for the treatment of metastatic tumors.

The pioneering work of Hugo Vankelecom set the stage for the identification of SCs in the pituitary gland, having roles in embryogenesis and in adult tissues, as well as in pituitary tumors (2, 3). Pituitary SCs and somatotroph cells express the proto-oncogene RET, which plays a role in cell survival. The study by Pradilla Dieste et al. systematically tackled the expression of the RET co-receptors GFRα 1,2,3, and 4 across the lobes and individual cell types of both rat and human pituitaries. Following extensive stainings, they could demonstrate that GFRα co-receptors are expressed in pituitary SCs at the niche in both species. The differential distribution of GFRαs is novel and provocative, indicating that some co-receptors' expression is retained in differentiated cells. This study suggests a potential involvement of GFRα co-receptors in maintaining stemness of the pituitary SC niche, and in directing proper lineage differentiation of adenopituitary cells, further supporting a role for RET/GFRα signaling in pituitary plasticity.

Although the isolation and characterization of cancer cells endowed with stemness properties has been achieved in several malignancies, the identification of tumor stem cells is not always child’s play. In their review Mantovani et al. summarize the current knowledge about the tumor stem cells (TSCs) from pituitary tumors, a relatively recent research line, which is born following the identification of normal stem cells in the adult pituitary gland (4), with the first study describing a population of cells in pituitary tumors presenting CSC properties published by Xu et al. in 2009 (5). However, contrasting and not fully convincing data have been reported about the *in vivo* tumorigenic capacity of TSCs in mouse models, and the lack of reproducible data on long-term and serial *in vivo* growth. Another important feature of TSCs is the therapy resistance, as observed in many other tumor models. Even in this context, different studies have highlighted that pituitary TSCs are sensitive to drugs currently used in clinical setting, thus questioning role of these cells in driving the pharmacological resistance observed in pituitary tumor patients (6, 7). On the same page regarding the clinical implication of pituitary adenoma stem cells, and the controversial findings obtained in their isolation and *in vitro*/*in vivo* experimentation is the group of Würth et al., who analyzed in depth the experimental settings used to isolate and characterize the pituitary adenoma stem cell population, with a focus on their specific activated signaling pathways associated with the regulation of stemness and EMT (*i.e.* Notch, Shh, Wnt and Hippo). These studies emphasize that, although consistent data are available regarding the existence of pituitary adenoma stem cells, there is still an urgent need to address some unsolved questions with future studies, to design effective therapeutic protocols for the treatment of chemoresistant/invasive pituitary tumors. Cancer stemness has been often associated with the acquisition of mesenchymal traits, driven by a process known as epithelial-to-mesenchymal transition (EMT) that sustains cancer cell progression, which has been observed in few endocrine cancers including pancreatic, prostate, thyroid, and pituitary cancers (8). In their review, Bhattacharya and Scimè analyze in detail the main metabolic processes involved in EMT, such as the shift in glucose utilization towards aerobic glycolysis and the changes in lipid metabolism, typical stem cell-like feature that characterize these transitions in pancreatic, prostate, thyroid, and pituitary cancers. Interestingly, acquisition of stem-like properties is not restricted to cancer cell only, but this process has also been described for fibroblast (cancer-associated fibroblasts) and adipose cells (cancer-associated adipose cells) in the context of tumor microenvironment (9). These findings highlight the metabolic plasticity in endocrine tumors of the cancer cells and of the tumor microenvironment components which can reciprocally induce re-acquisition of stem-like properties supporting cancer progression.

All these findings highlight the controversial origin and the key role of CSCs in different steps of ETs’ progression, thus underlining the clinical importance of their study for the development of innovative target therapies, to improve the quality of life of ETs’ patients. Nonetheless, further studies are urgently needed to solve the existing controversial findings, and to develop novel and reliable *in vitro*/*in vivo* models to increase the scientific soundness and reproducibility of the obtained results.

## Author Contributions

SF, NP, ML, and GS participated in the design of the manuscript, analyzed the bibliographic data, collected them, and drafted the manuscript. ML and GS critically revised the manuscript. All authors contributed to the article and approved the submitted version.

## Funding

This work was supported by funding from German Research Foundation (DFG) project # 391523415-SFB824 (B8) and project # 314061271-TRR 205 (B11), German Cancer Aid (# 70112383), and Wilhelm Sander Stiftung foundation (# 2017.012.1) to NP; AIRC-CRF Multi-user Equipment Program ID 19515, and AIRC Investigator Grant IG2015 ID17691 to ML; AIRC IG (21445), and PRIN 830 2017WNKSLR to GS.

## Conflict of Interest

The authors declare that the research was conducted in the absence of any commercial or financial relationships that could be construed as a potential conflict of interest.
